# Arsenobetaine amide: a novel arsenic species detected in several mushroom species

**DOI:** 10.1007/s00216-024-05132-z

**Published:** 2024-01-16

**Authors:** Martin Walenta, Andrea Raab, Simone Braeuer, Lorenz Steiner, Jan Borovička, Walter Goessler

**Affiliations:** 1 Institute of Chemistry, Analytical Chemistry, University of Graz, Universitaetsplatz 1, 8010 Graz, Austria; 2 Faculty of Chemistry, Institute of Analytical Chemistry, University of Vienna, Waehringer Strasse 38, 1090 Vienna, Austria; 3 Institute of Chemistry, Inorganic Chemistry, University of Graz, Universitaetsplatz 1, 8010 Graz, Austria; 4 Institute of Geology of the Czech Academy of Sciences, Rozvojová 269, 16500 Prague 6, Czech Republic; 5 Nuclear Physics Institute of the Czech Academy of Sciences, Hlavní 130, 25068 Husinec-Řež, Czech Republic

**Keywords:** Mushrooms, *Ramaria sanguinea*, Arsenic speciation, Trimethylarsonioacetamide, HPLC-ICPMS, HR ESI-MS

## Abstract

The total arsenic mass fraction as well as the arsenic speciation were studied in four different mushroom species with inductively coupled plasma mass spectrometry and high-performance liquid chromatography coupled to inductively coupled plasma mass spectrometry, respectively. Arsenic mass fractions detected in the mushrooms were covering a range from 0.3 to 22 mg As kg^−1^ dry mass. For the arsenic speciation, species like arsenobetaine, inorganic arsenic, or dimethylarsinic acid were found, which are commonly detected in mushrooms, but it was also proven that the recently discovered novel compound homoarsenocholine is present in *Amanita muscaria* and *Ramaria sanguinea*. Moreover, a previously unidentified arsenic species was isolated from *Ramaria sanguinea* and identified as trimethylarsonioacetamide, or in short: arsenobetaine amide. This new arsenical was synthesized and verified by spiking experiments to be present in all investigated mushroom samples. Arsenobetaine amide could be an important intermediate to further elucidate the biotransformation pathways of arsenic in the environment.

**Graphical Abstract:**

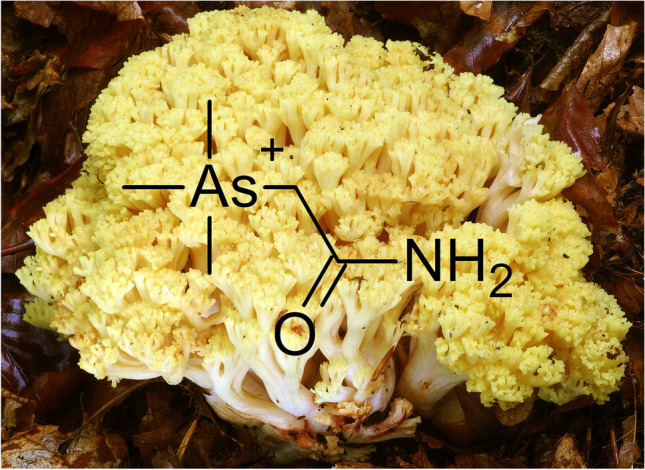

**Supplementary Information:**

The online version contains supplementary material available at 10.1007/s00216-024-05132-z.

## Introduction

**Fig. 1:**
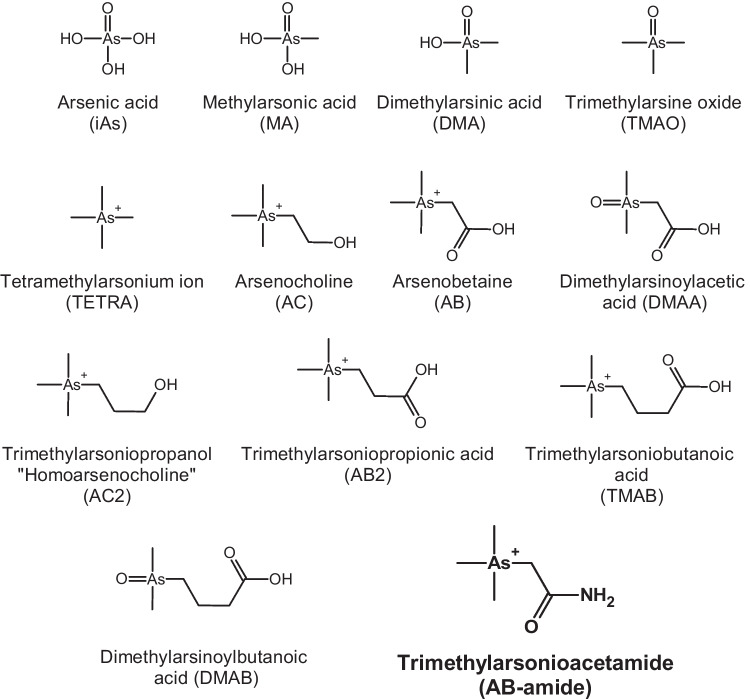
Arsenic species relevant for this publication

In a study about the global fungal diversity from 2021, including published datasets of roughly a million different fungal species, the total number of fungal species present in the world was estimated to be over six million [1]. Some of these fungi, specifically mushrooms, are known to have an extremely diverse arsenic speciation in their fruiting-bodies. In contrast to other organisms, it is almost impossible to predict the arsenic speciation in mushrooms just by comparison with similar mushroom species [2–4]. Some mushrooms contain mainly inorganic arsenic (iAs, in the form of arsenate and arsenite), dimethylarsinic acid (DMA), and/or methylarsonic acid (MA) (Fig. 1) which are usually found in terrestrial organisms [5–7]. However, the most often found main arsenic species in mushrooms is arsenobetaine (AB), which is commonly found in marine animals [8]. Furthermore, traces of arsenocholine (AC), the tetramethylarsonium ion (TETRA), trimethylarsine oxide (TMAO) (Fig. 1), and unidentified arsenic species can be detected in mushrooms and sometimes even as the main species [3, 4, 7, 9, 10].

It is still unclear whether mushrooms are able to metabolize arsenic or if they simply take up different arsenic species from the substrate or if other microorganisms are involved in these processes. A number of studies tried to investigate this question, but no definitive answer could be found [11, 12]. To further elucidate the pathway of arsenic and its possible biotransformation, identifying intermediate compounds could be of valuable help. Therefore, it is important to identify previously unknown arsenic species [10, 13, 14], which will hopefully help us to better understand the reason behind the diversity of arsenic species in mushrooms.

In a previous study on edible mushrooms, *Boletus edulis* (king bolete) and *Macrolepiota procera* (parasol mushroom) proved to exhibit a complex distribution of arsenic species, including some hitherto not identified arsenic species [15]. Other good candidates for interrogating potential intermediates would be the famous *Amanita muscaria* (fly agaric), which was already reported to contain several unidentified arsenic species [4, 9], as well as the *Ramaria* genus, since they also possess a complex arsenic speciation profile and tend to take up more arsenic than any of the mushrooms mentioned above [10]. This higher mass fraction of arsenic would simplify the process of isolating a potentially new arsenic compound and identifying it.

In this study, we investigated the arsenic speciation in *B. edulis*, *M. procera*, *A. muscaria*, and *Ramaria sanguinea*. We employed high-performance liquid chromatography (HPLC) coupled to inductively coupled plasma mass spectrometry (ICPMS) to unravel the arsenic speciation of these mushrooms with some unidentified signals and aimed to identify one of the unknown arsenic species. Therefore, we thoroughly enriched and purified the unknown compound by collecting fractions of mushroom extracts by HPLC and freeze-drying the pooled collection. Finally, we identified a novel nitrogen-containing arsenic species using HPLC coupled to high-resolution mass spectrometry. This new arsenic compound will give us a new perspective in the current understanding of the arsenic speciation in the environment and add to the knowledge about the biotransformation ways of arsenic.

## Materials and methods

### Mushroom collection and processing

For this study, we used eleven individual mushroom fruiting-bodies collections as samples, which were collected in different regions of Austria and the Czech Republic. From three of the samples (*B. edulis* 1 + 4 and *M. procera* 1), the arsenic speciation was already published and discussed in a previous publication [15]. We investigated mushroom samples from non-contaminated as well as arsenic-contaminated regions (summarized in Supplementary Information Table S1). These samples were selected, because preliminary analysis of these mushroom species showed them to have a very diverse arsenic speciation profile and many unknown arsenic species with similar chromatographic behavior. The sample handling, sample preparation, and determination of the total arsenic content as well as the determination of the common water-soluble arsenic species are described in detail elsewhere [15].

Briefly, the samples were directly brought to the laboratory, where they were carefully cleaned before freeze-drying (Christ, Osterode am Harz, Germany) and homogenization (ultra-centrifugal mill ZM200, 1 mm titanium sieve, Retsch GmbH, Haan, Germany).

The freeze-dried mushrooms were digested in triplicates with nitric acid (p.a., ≥65%; Carl Roth, Karlsruhe Germany, sub-boiled in-house) in a microwave-heated autoclave (Ultraclave IV, MLS GmbH, Leutkirch, Germany; temperature ramp up to 250 °C, pressure up to around 100 bar) and investigated with ICPMS (7700x, Agilent Technologies, Waldbronn, Germany) for the determination of the total arsenic mass fraction. The performance of the instrument can be found in Table S2. For quality control, the Standard Reference Material® (SRM) 1573a (Tomato Leaves, NIST, Gaithersburg, USA), the certified reference material (CRM) IPE-120 (*Agaricus bisporus*, WEPAL, Wageningen, Netherlands), and the SRM® 1643f (Trace elements in water, NIST, Gaithersburg, USA) as well as blanks consisting of ultrapure water (18.2 MΩ*cm, Merck Millipore, Bedford, USA) were prepared and measured with the samples. To control the stability of the measurement, germanium was added as internal standard via a t-piece before the nebulizer.

For arsenic speciation analysis, dried mushroom samples were extracted in triplicates with ultrapure water and investigated by coupling HPLC (1200, Agilent Technologies) to ICPMS with carbon dioxide as optional gas, added via optional gas line, to compensate for the carbon enhancement effect. An anion-exchange column (PRP-X100) with an aqueous 20 mM phosphate buffer (pH 6.0) and a cation-exchange column (Reprosil-XR 300 SCX) with an aqueous 10 mM pyridine solution (pH 2.3) were applied to detect and quantify iAs, DMA, MA, AB, TMAO, AC, and TETRA using an external calibration containing these arsenic species. The results for the quality control of all measurements were in good accordance with the certified or published data [6, 13, 15] (Table S3 and S4). The total arsenic mass fraction of the extracts was determined by diluting the samples 1+9 with 10% v/v nitric acid and directly measuring it with ICPMS.

A quadrupole time of flight MS (6546, Agilent Technologies) was used for high-resolution electrospray ionization mass spectrometry (HR ESI-MS) measurements in positive mode (Table S5). It was coupled to HPLC equipped with a cation-exchange column (Zorbax 300-SCX, 4.6 × 150 mm, 5 µm, Agilent Technologies) at 30 °C, 30 mM ammonium formate, pH 5.0 as mobile phase, and a flow rate of 0.6 mL min^−1^.

All ^1^H and ^13^C NMR spectra were recorded in deuterated dimethyl sulfoxide (DMSO-d6) on an Avance III 300 MHz spectrometer (Bruker Corporation, Billerica, USA) at 300 K and the spectra were processed using the MestreNova software (Mestrelab Research).

## Results and discussion

The total arsenic mass fraction in the different mushroom samples ranged from 0.28 ± 0.01 mg kg^−1^ dry mass (dm) found in a *B. edulis* sample to 22 ± 2 mg kg^−1^ dm found in an *A. muscaria* sample (Table 1). The extraction efficiency was determined by comparing the total arsenic mass fraction found after digestion with nitric acid, with the total arsenic mass fraction found in the aqueous extracts. Across all the analyzed mushrooms, the extraction efficiency was 77 ± 11 % extracting the water-soluble arsenicals. For column recovery, the cation-exchange chromatogram was integrated at the baseline over the whole time span and quantified via compound independent calibration using AB for calibration. This arsenic mass fraction recovered in the chromatogram was compared to the total arsenic mass fraction found directly in the extracts to give a column recovery of 99 ± 6 %.

**Table 1:** Mass fractions of total arsenic and extracted arsenic in the mushrooms [mg As kg^−1^ dry mass] and some of the detected arsenic species [% of total arsenic]. For Sample ID and more information, see Table S6. *n*=3 for total As and As speciation analysis for all samples, except *B. edulis* 2 (*n*=3 for total As, *n*=1 for As speciation analysis, because of insufficient sample material). *UNK*, sum of detected unknown As species

Sample	Total As [mg kg^−1^]	Extr. As [mg kg^−1^]	DMA [%]	AB [%]	AC [%]	AC2 [%]	UNK [%]
*R. sanguinea*	17.2 ± 0.2	14.0 ± 0.2	0.17 ± 0.01	33 ± 1	14 ± 1	7.6 ± 0.1	15 ± 1
*A. muscaria* 1	22 ± 2	21 ± 2	3.6 ± 0.1	48 ± 1	3.5 ± 0.2	2.6 ± 0.2	1.5 ± 0.1
*A. muscaria* 2	0.74 ± 0.01	0.61 ± 0.01	10 ± 1	44 ± 1	3.0 ± 0.2	1.8 ± 0.1	0.4 ± 0.1
*A. muscaria* 3	1.04 ± 0.02	0.87 ± 0.02	6.6 ± 0.2	46 ± 1	2.2 ± 0.2	1.7 ± 0.1	1.5 ± 0.1
*A. muscaria* 4	2.03 ± 0.04	1.3 ± 0.1	6.8 ± 0.7	37 ± 3	1.1 ± 0.1	0.7 ± 0.1	0.9 ± 0.2
*B. edulis* 1	1.02 ± 0.06	0.70 ± 0.02	14 ± 1	10 ± 2	3.5 ± 0.2	<0.2	23 ± 3
*B. edulis* 2	1.75 ± 0.03	0.96	1.8	8.7	3.1	<0.2	17
*B. edulis* 3	0.54 ± 0.02	0.41 ± 0.01	7.5 ± 1.1	18 ± 2	7.4 ± 0.8	<0.4	11 ± 2
*B. edulis* 4	0.28 ± 0.01	0.20 ± 0.01	6.6 ± 0.5	16 ± 2	1.7 ± 0.1	<0.7	26 ± 4
*M. procera* 1	0.89 ± 0.02	0.74 ± 0.04	11 ± 1	40 ± 2	2.1 ± 0.2	<0.2	6.4 ± 0.8
*M. procera* 2	4.5 ± 0.2	3.7 ± 0.1	0.5 ± 0.1	64 ± 2	1.5 ± 0.1	<0.1	4.3 ± 0.8

AB was the main arsenic species found in all mushroom samples, except the sample *B. edulis* 1, which had DMA as the main arsenic species. However, this mushroom species is known to contain some unidentified arsenicals and has no clear trend to a certain arsenic species [15]. We also found different amounts of iAs, MA, TMAO, TETRA, trimethylarsoniopropionic acid (AB2), and homoarsenocholine (AC2) (Fig. 1). These arsenic species were confirmed by spiking and co-chromatography. The most important results are given in Table 1. Mass fractions of all detected arsenic species can be found in Table S6. As a side note, we now proved, as already speculated in a previous report, that AC2 is indeed present in *A. muscaria* and can also be detected in the *R. sanguinea* [10].

**Fig. 2:**
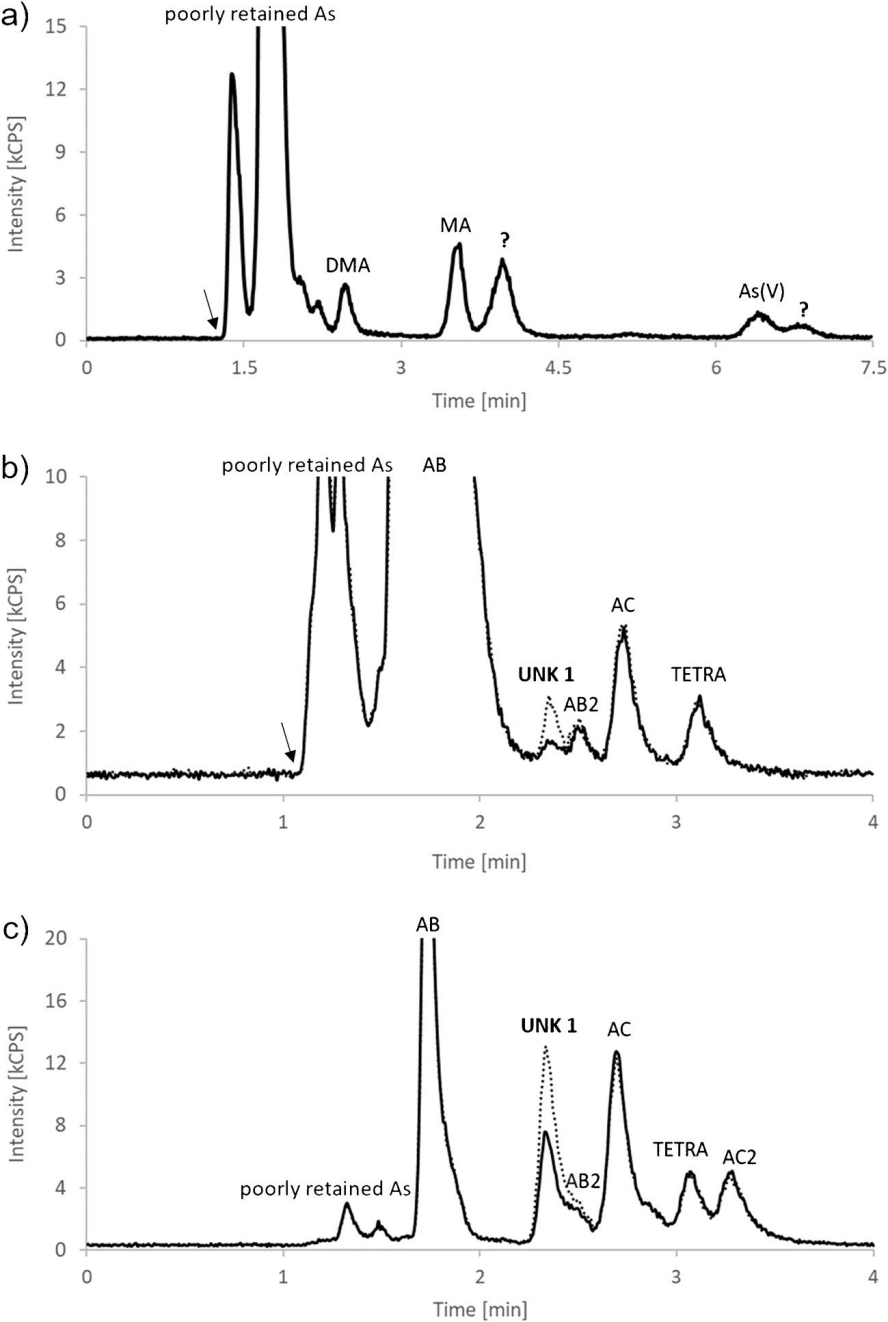
Typical anion-exchange (**a**) and cation-exchange (**b**) chromatogram of a *Macrolepiota procera* 2 extract and a cation-exchange chromatogram (**c**) of a *Ramaria sanguinea* extract. Dotted lines show the extracts spiked with synthetic AB-amide. The small arrow indicates the void time of the method used

Several unassigned peaks were found, especially in the cation-exchange chromatograms (see Fig. 2). With spiking experiments, we excluded dimethylarsinoylbutanoic acid (DMAB) and trimethylarsoniobutanoic acid (TMAB) as possible candidates (Fig. 1). Furthermore, addition of hydrogen peroxide to the sample proved that no known thio-arsenic compound was present, and that the unknown compounds likely contained arsenic in oxidation state +5.

Our attention was mainly attracted by unknown compound UNK 1, eluting between TMAO and AB2, because it was present in detectable amounts in all investigated samples. Its mass fraction was highest in *R. sanguinea*, accounting for 1.65 ± 0.01 mg As kg^−1^ dm. This promised a good possibility to clarify the structure of UNK 1.

To further enrich the concentration of UNK 1 in solution, we adapted the cation-exchange method to an ammonium formate buffer instead of pyridine, which would sublime via freeze-drying. Fractions mainly containing UNK 1 were obtained with this adapted method by injecting an aqueous extract of the mushroom (*R. sanguinea*) multiple times onto the cation-exchange column and collecting the effluent around the retention time of UNK 1.

**Fig. 3:**
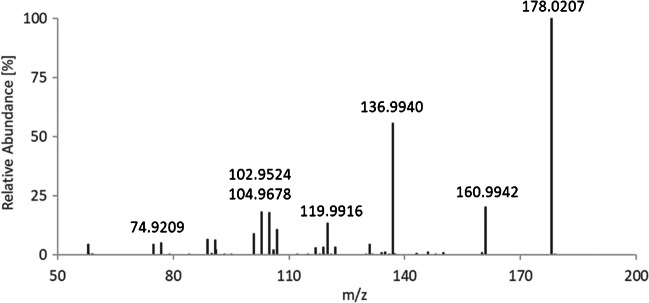
Mass spectrum of HR ESI-MS of the UNK 1 (AB-amide) fraction collected

After the collection, the fractions were pooled, frozen, and freeze-dried, before the residue was redissolved in a small volume of ultrapure water. The presence of UNK 1 in the isolate was confirmed with HPLC-ICPMS and spiking it to the *R. sanguinea* extract. By introducing the isolate to HR ESI-MS, we detected at the corresponding elution time, as specified with HPLC-ICPMS, a molecule with an exact mass of *m/z* of 178.0207 (Δ= −0.2 ppm) and a proposed sum formula of C_5_H_13_AsNO^+^. Characteristic fragments of the compound under fragmentation experiments can be seen in Fig. 3.

The *m/z* 161 represents an ammonia loss, *m/z* 137 corresponds to the loss of vinylamine, *m/z* 120 is a charged Me_3_As, *m/z* 103/105 is Me_2_As-2H and Me_2_As, and *m/z* 75 is the charged As. Similar fragments can be found for the fragmentation of other arsenicals [16], but the nitrogen group’s exact location in the molecule could not be determined with the obtained HR ESI-MS data. We assumed that it would be preferably at the same carbon as the oxygen and could possibly be a derivate of AB, so we used an adapted literature procedure for AB [17], to synthesize trimethylarsonioacetamide, which we called arsenobetaine amide (“AB-amide”, Fig. 1).

**Fig. 4:**
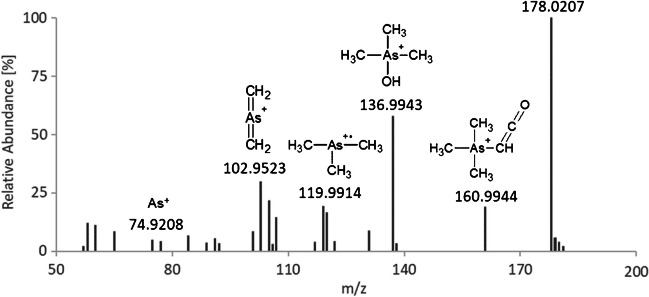
Mass spectrum of HR ESI-MS of the synthesized AB-amide and potential structures [16] of the fragments detected

In a nitrogen flushed Schlenk flask equipped with a stirring bar, trimethylarsine (1 mL, 9.4 mmol) was added to 10 mL of dry, deoxygenated toluene. The flask was cooled to 0 °C in an ice bath. 2-Bromoacetamide (1.38 g, 7.2 mmol) was added to the solution and the mixture was stirred at room temperature for 48 h. The formed solids were collected by filtration in air and washed with acetone to afford 473 mg (25 %) of AB-amide as colorless crystals. The purity and structure were confirmed with ^1^H and ^13^C NMR experiments and HR ES-MS (see Fig. 4).

- ^1^H NMR (300 MHz, DMSO-d6): δ = 7.91 (s, NH, 1H), 7.48 (s, NH, 1H), 3.58 (s, CH_2_, 2H), 1.90 (s, 3 x Me, 9H);
- ^13^C NMR (75 MHz, DMSO-d6): δ = 167.9, 32.9, 8.7.

The results were in accordance with our findings of UNK 1, and a successful spiking of the standard to all mushroom samples analyzed further supported that UNK 1 is indeed AB-amide.

To our knowledge, the synthesis of AB-amide has not been published as of now, and the molecule was never reported in any previous study. Additionally, even the analogous ammonium-compound betaine-amide itself is hardly ever explored in scientific literature [18]. That these compounds have almost never been studied or mentioned before is particularly interesting, especially since their chemical structures are so similar to better-studied derivatives such as the arsenic-containing AB [19–21] and the nitrogen analogue glycine betaine [21, 22].

Currently there are different pathways proposed how AB is synthesized in marine or terrestrial environments [20, 23, 24]. For most of them, the final biotransformation step to achieve AB is the oxidation of AC or the methylation of dimethylarsinous acid. As AB-amide is a derivate of AB, it is possible that the amide could fit into previously reported biotransformation pathways of AB as a stable intermediate step, as a side reaction product or that AB-amide is a result of a different starting or intermediate compound. This last option would imply that other nitrogen-containing arsenic species such as the amine analogue of AC or the amide analogue of DMAA may also be present. A clear proof to explain how AB-amide fits into the whole biotransformation of arsenic in these mushrooms is not existing and further experiments will be needed for clarification.

In general, reports of nitrogen-containing arsenic species are rarely seen. Historically, some nitrogen-containing arsenic species were developed and applied as drugs, such as arsanilic acid (Atoxyl) against sleeping sickness (trypanosomiasis) and arsphenamine, better known as Salvarsan, as treatment of syphilis [25, 26]. To our knowledge, in natural samples, only the nitrogen-containing arsinothricin [27], which is produced by a rice bacterium, and a handful of arsenosugars with nitrogen-containing side groups were previously identified [28, 29].

## Conclusion and outlook

In this report, AB-amide, a nitrogen-containing arsenic species, was identified for the first time in a natural sample. In the investigated mushrooms, AB-amide accounted for 0.15 to 9.6% of total As or in mass fractions 0.002 to 1.7 mg As kg^−1^ dm. This discovery sheds a new light on the understanding of the biogeochemical pathway of arsenic in the environment. Further identification of other arsenic species in the environment, especially nitrogen-containing ones, would help to better understand the potential role of AB-amide. Looking at chromatograms of previous publications, it can be speculated that “unknown” arsenic species in mushrooms [4, 10] and potentially even in sea snails [30] could indeed be AB-amide. In the future, additional experiments are needed to prove this claim, but if it is verified that AB-amide is present in sea snails, this compound would not only play a role in the biotransformation pathway of arsenic in the fungal kingdom but also in others. This highlights the importance of identifying new arsenic compounds even more to complete our knowledge of the arsenic biotransformation pathways.

### Supplementary Information

Below is the link to the electronic supplementary material.

Supplementary file1 (XLSX 30 KB)

## Data Availability

The data that support this study is shared in the supplementary material. Further data will be shared upon reasonable request to the corresponding author.
